# Modeling outbreak data: Analysis of a 2012 Ebola virus disease epidemic in DRC

**DOI:** 10.11145/j.biomath.2019.10.037

**Published:** 2019-10-15

**Authors:** Boseung Choi, Sydney Busch, Dieudonné Kazadi, Benoit Ilunga, Emile Okitolonda, Yi Dai, Robert Lumpkin, Omar Saucedo, Wasiur R. KhudaBukhsh, Joseph Tien, Marcel Yotebieng, Eben Kenah, Grzegorz A. Rempala

**Affiliations:** * Department of National Statistics, Korea University Sejoung Campus Sejoung, Republic of Korea; † Department of Mathematics, Augsburg College Minneapolis, MN, USA; †† Ministry of Health, Democratic Republic of the Congo; ‡‡ School of Public Health, University of Kinshasa Kinshasa, Democratic Republic of the Congo; ‡ Division of Biostatistics, College of Public Health, The Ohio State University, Columbus, OH, USA; § Department of Mathematics, The Ohio State University, Columbus, OH, USA; ¶ Mathematical Biosciences Institute, The Ohio State University, Columbus, OH, USA; ‖ Division of Epidemiology, College of Public Health, The Ohio State University, Columbus, OH, USA

**Keywords:** parameter estimation, branching process, Markov Chain Monte-Carlo methods, survival dynamical system

## Abstract

We describe two approaches to modeling data from a small to moderate-sized epidemic outbreak. The first approach is based on a branching process approximation and direct analysis of the transmission network, whereas the second one is based on a survival model derived from the classical SIR equations with no explicit transmission information. We compare these approaches using data from a 2012 outbreak of Ebola virus disease caused by *Bundibugyo ebolavirus* in city of Isiro, Democratic Republic of the Congo. The branching process model allows for a direct comparison of disease transmission across different environments, such as the general community or the Ebola treatment unit. However, the survival model appears to yield parameter estimates with more accuracy and better precision in some circumstances.

## I. Introduction

On August 1, 2018, the Ministry of Health of the Democratic Republic of the Congo (DRC) reported an outbreak of Ebola virus disease (EVD) in North Kivu Province. At the time of writing about one year later, confirmed and probable cases have been reported in nine health zones of North Kivu and Ituri provinces (including the provincial capital city of Goma), threatening further spread of the epidemic into neighboring provinces and the countries of Uganda and Rwanda. The current outbreak area is roughly 780 miles away from Equateur province, where an earlier Ebola outbreak was reported in May 2018. This persistent reoccurrence of Ebola in the DRC as well as elsewhere in Africa is a reminder that another large pandemic like the 2013–2016 West African Ebola epidemic remains possible. To control future outbreaks and to better understand patterns of transmission in households and at health care facilities, it is essential to carefully analyze well-documented historic data from past outbreaks.

The current paper is concerned with modeling data from a small 2012 Ebola virus disease (EVD) outbreak caused by *Bundibugyo ebolavirus* (BDBV) in the Isiro municipality in DRC. The interesting feature of this dataset is that it includes partial contact information on Ebola cases treated either in the community or in healthcare facilities, which allows for network-based inference. Despite the fact that such inference has been an extremely active area of research in the past 20 years [1]–[5], there have been relatively few well documented historical datasets from real epidemics. For our purpose of analyzing a relatively small network, we here apply the *edge-based* approach of Miller and Volz [15]–[17] and compare it with the recently proposed simple non-network *survival dynamical system* model [18].

The paper is organized as follows. In the remainder of this section we give some basic background information on the Ebola virus and describe the Isiro outbreak dataset to be analyzed. In Sections 2 and 3, we outline the proposed statistical models, use them to analyze the Isiro outbreak data, and describe the results. In Section 4, we offer some brief concluding remarks.

### A. Ebola virus disease

EVD in humans is a severe hemorrhagic fever caused by four of the five viruses of the genus *Ebolavirus* in the viral family *Filoviridae* [19]. The virus was initially characterized in 1976 during an outbreak in DRC (known then as Zaire) and has subsequently caused at least 26 additional outbreaks in human populations [20], [21]. Since 2000, there have been 8 recorded outbreaks in the DRC. The ongoing outbreak in the North Kivu province is the most serious to date, and Maganga et al. [22] describe an earlier serious outbreak that occurred in the Boende region of Équateur Province in 2014. Data is available in the literature for at least 4 out of these 8 outbreaks, although these data are not considered fully reliable because the majority of the outbreaks occurred in remote areas. Although EVD outbreaks in DRC have been limited to fewer than 500 cases to date, the potential for dangerous spread across the African continent was demonstrated by the 2013–2016 West African Ebola epidemic, which resulted in more than 28,600 cases and 11,313 deaths across ten countries [23].

The introduction of the virus into human communities is likely the result of sporadic zoonotic events. Several species of fruit bats native to areas endemic to Ebola have been implicated as the natural reservoir for the disease [19]. Upon infection, the virus will typically incubate for a period of two to 21 days [23]. After this period, the typical clinical presentation is a mix of severe symptoms that may include fever, nausea, diarrhea, vomiting, chest pain, dyspnea, cough, ocular edema, hypotension, conjunctivitis, headaches, coma, and hemorrhaging [24].

Human-to-human Ebola transmission is believed to occur via close contact with infected bodily fluids and, therefore, individuals such as family members of cases and healthcare workers have significantly increased risk of infection [25]–[28]. In addition, viral load (which changes throughout the course of illness and is at the highest level immediately after death) is known to impact the probability of transmission [29], [30]. Transmission dynamics are further complicated by the fact that an infectious individual’s contacts may vary throughout disease progression. Accounting for these dynamics through contact tracing and network analysis is vital to understanding the disease, as studies of previous outbreaks have noted disproportionately high rates of infection among women, healthcare workers, and family members [23], [25], [27], [31].

Practical strategies have been implemented to reduce the risk of transmission, including barrier nursing methods, safe burial practices, and the creation of isolation units within treatment centers [31], [32]. Nevertheless, the scope of the 2013–2016 West African Ebola epidemic suggests that a deeper quantitative understanding of these dynamics might be needed to control transmission more effectively. While an effective vaccine is now available [33], the question of how and when to most effectively use available resources during an ongoing epidemic remains in need of further quantitative study.

Prior to the West African Ebola epidemic, only a handful of mathematical models for Ebola had been studied [34]–[37]. Chowell et al. [34] and Lekone and Finkenstadt [35] used an SEIR framework to determine the effect of interventions on the 1995 DRC and 2000 Uganda outbreaks. Legrand et al. [36] formulated a stochastic compartmental model that accounted for transmission in several epidemiological settings by introducing compartments for hospitalized individuals and dead Ebola cases, who can transmit the disease during funerals.

The West African epidemic highlighted the critical need for a better understanding of the Ebola transmission dynamics and potential control measures. Consequently, there has been an out-pouring of Ebola models including deterministic compartmental models [38]–[40], stochastic models [41]–[44] and multi-type branching process models [45]. Many of these adapted the SEIHRF framework, which includes hospital and funeral compartments [26], [46]–[48].

Recent work has considered spatial aspects of Ebola virus transmission and the effect of clustering in the population. The spatiotemporal spread of Ebola has been studied with a county-level multi-patch model employing mobile data [41], with spatial individual-based models for international spread [43], and transmission between households [44]. Scarpino et al. [26] used a phylodynamic model to reconstruct chains of transmission for cases occurring in Sierra Leone in June 2014. Their fit of an SEIR model with the Rand-style [1] pair approximation gave evidence for the presence of clustering within the population. The same pair approximation was employed by Wells et al. [46] in their investigation of the effectiveness of case isolation and ring vaccination. Their model had 10 compartments and required 65 equations after closure at the level of pairs.

### B. Isiro EVD outbreak data

**Fig. 1:**
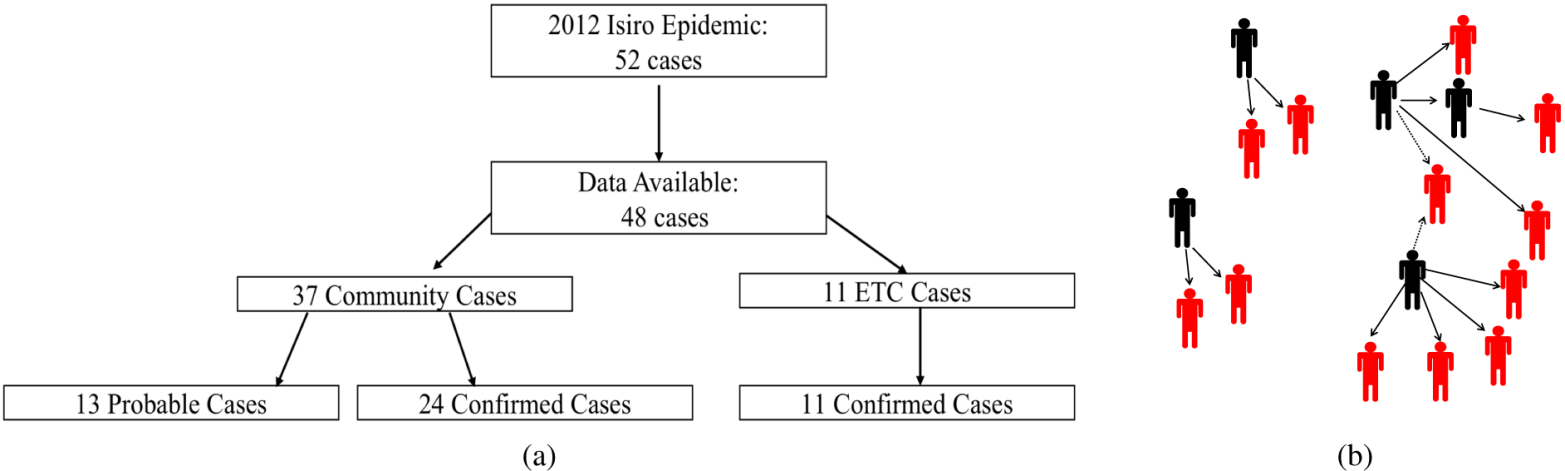
2012 Isiro EVD data and model. Panel (a): Summary of available Isiro cases used in current analysis of the transmission dynamics in the community and ETC. This data is a subset of 52 cases described in [49]. Panel (b): Example of transmission data reconstructed from the Isiro outbreak files. Dark figures represent primary cases and secondary cases who infected others. All others represent infected who did not transmit. All cases of transmission ambiguity (multiple in-arrows) were resolved uniformly at random.

Isiro is a municipality in the north-east part of DRC that is the capital of Haut-Uele district. It is situated between equatorial forest and savannah. The Isiro dataset contains information about the 2012 EVD outbreak caused by *Bundibugyo ebolavirus* (BDBV). In total, there were 62 cases of infection listed as either probable or confirmed with 52 of them having proper clinical information allowing for a more detailed study. As shown in Figure 1(a), these were divided into community cases and Ebola treatment center (ETC) cases based on the source of the infecting contact. Among community cases, there was over-representation of females (85.3%) and of individuals aged 15–54 years (82.4%). The mean duration of EVD was 18 days, and the mean incubation period was 11.3 days [49].

We were able to obtain additional information concerning contacts for most individual cases from the DRC Ministry of Health. However, unlike traditional contact tracing, these additional records contained a list of potentially infecting individuals for each case. Using this information, we were able to track the likely number of people each case infected as well as the overall contact network (for most of the EVD cases) in both the community and the ETC. To refine the transmission network, we used occupation and other socioeconomic information as available. Of the 52 documented infections in the Isiro epidemic, clinical documentation of contacts among 48 cases (17 probable, 31 confirmed) could be retrieved. Out of these, there were 37 community cases^1^ (13 suspected or probable and 24 confirmed) who never reached the ETC. The 11 cases that reached the ETC were all confirmed as EVD [49].

## II. Statistical models

In this paper, we consider two different statistical models for the Isiro EVD outbreak. The first one is based on a branching process approximation of the virus transmission network, which appears especially appropriate for directly comparing outbreak parameters in different environments such as the village community and the ETC. The second model is based on a survival analysis approach that assumes homogenous contact patterns among susceptibles and infectives. This assumption is more likely to be appropriate among community cases than ETC cases.

### A. Branching model

#### 1) Primary cases:

For a primary (or index) case *i* represented by a node of degree *d*_*i*_, the distribution of the number of secondary infections created by *i* (say, *X*_*i*_) conditionally on the degree and the infection period (say, *t*_*i*_) is given by

(1) $$P\left(X_{i}=x_{i}|t_{i},d_{i}\right)=\left(\begin{array}{c} d_{i}\\ x_{i} \end{array}\right)p_{t_{i}}^{x_{i}}{\left(1-p_{t_{i}}\right)}^{d_{i}-x_{i}}.$$

We see therefore that the distribution function of *X*_*i*_ is binomial with *d*_*i*_ trials and the probability of success $p_{t_{i}}$. Under the assumption that infectious contacts follow a Poisson process with rate *β*, the probability of a successful infection of a given neighbor by case *i* before time *t*_*i*_ is

$$p_{t_{i}}=1-e^{-\beta t_{i}}.$$

The infectious period (i.e., time from symptom onset to removal) for each case is assumed to follow an exponential distribution with rate *γ*, so its density is

(2) $$f\left(t_{i}\right)=\gamma e^{-\gamma t_{i}}.$$

For the *i*-th index case the joint conditional probability distribution of (*x*_*i*_, *t*_*i*_) given *d*_*i*_ is the product of (1) and (2):

$$\pi_{d_{i}}\left(x_{i},t_{i}\right)=P\left(X_{i}=x_{i}|t_{i},d_{i}\right)f\left(t_{i}\right)\;=\left(\begin{array}{c} d_{i}\\ x_{i} \end{array}\right)\gamma {\left(1-e^{-\beta t_{i}}\right)}^{x_{i}}e^{-\left(\beta \left(d_{i}-x_{i}\right)+\gamma \right)t_{i}}.$$

Without any prior information on the degree *d*_*i*_ of the *i*-th index case, the unconditional joint probability distribution of the number of infections and the infection period is then given by

(3) $$f\left(x_{i},t_{i}\right)=\underset{d_{i}\geq x_{i}}{\sum }q_{d_{i}}\pi_{d_{i}}\left(x_{i},t_{i}\right)$$

where *q*_*d*_ is the probability of degree *d*. Here, we consider the Poisson distribution with mean *λ*,which appears to fit well the transmission network for the Isiro epidemic. With this degree distribution, the final form of (3) admits the following closed-form expression parametrized by the triple $\theta =(\beta ,\gamma ,\lambda )\in {\mathbb{R}}_{>0}^{3}$:

(4) $$f_{\theta }\left(x_{i},t_{i}\right)=\;\underset{d_{i}\geq x_{i}}{\sum }\frac{\lambda^{d_{i}}e^{-\lambda }}{d_{i}!}\left(\begin{array}{l} d_{i}\\ x_{i} \end{array}\right)\gamma {\left(1-e^{-\beta t_{i}}\right)}^{x_{i}}e^{-\left(\beta \left(d_{i}-x_{i}\right)+\gamma \right)t_{i}}\;=\frac{{\left[\lambda \left(1-e^{-\beta t_{i}}\right)\right]}^{x_{i}}}{x_{i}!}e^{-\lambda \left(1-e^{-\beta t_{i}}\right)}\gamma e^{-\gamma t_{i}}.$$

#### 2) Secondary cases:

Consider now the joint distribution of the number of infections and the infection period for a secondary (i.e., non-index) case. For tractability, we assume the random network follows the configuration model (CM), so edges are formed uniformly at random (excluding multiple edges and self-loops) given the degrees of all nodes. For such networks, we define a *secondary case* as an individual (node) to whom the infection was successfully transmitted (see also [50]). Note that since by definition the secondary case has one infecting neighbor, its degree available for further infection decreases by one. This decreased degree is often referred to as the *excess degree* in the literature.

For secondary cases, we can modify the primary case model by replacing the degree with the excess degree. Let $q_{d_{j}}^{\prime }$ denote the excess degree probability for a secondary case with degree *d*_*j*_ > 0, and let *μ* ∈ (0,∞) be the mean of the degree distribution. Then it is known ([51]) that for the CM network

$$q_{d_{j}}^{\prime }=\frac{d_{j}q_{d_{j}}}{\mu }.$$

In the case of the Poisson degree distribution, *λ* = *μ* and it is easy to check that

(5) $$q_{d_{j}}^{\prime }=q_{d_{j}-1}.$$

Let $x_{j}^{\prime }$ and $t_{j}^{\prime }$ be the number of infections and the infection period for the secondary case *j*. Equations (3) and (5) give us

(6) $$f\left(x_{j}^{\prime },t_{j}^{\prime }\right)=\underset{d_{j}>x_{j}^{\prime }}{\sum }q_{d_{j}}^{\prime }\pi_{d_{j}-1}\left(x_{j}^{\prime },t_{j}^{\prime }\right)\;=\underset{d_{j}>x_{j}^{\prime }}{\sum }q_{d_{j}-1}\pi_{d_{j}-1}\left(x_{j}^{\prime },t_{j}^{\prime }\right)\;=f_{\theta }\left(x_{j}^{\prime },t_{j}^{\prime }\right),$$

where *f*_*θ*_ is given by (4).

For known *θ* = (*β*, *γ*, *λ*), we can calculate the key characteristic of an epidemic known as the *basic reproduction number* $\left(R_{0}\right)$. It may be interpreted as the average number of secondary infections caused by a primary case. For an arbitrary degree distribution the definition of $R_{0}$ for CM graph is given, for instance, in [52]. For the Poisson degree distribution with exponential infectious periods, we have simply

(7) $$R_{0}=\frac{\beta \lambda }{\beta +\gamma }.$$

#### 3) Likelihood and estimation:

Suppose the observed numbers of infections and infectious periods for *m* primary cases are ***x*** = {*x*_1_*, x*_2_, …, *x*_*m*_} and ***t*** = {*t*_1_, *t*_2_, …, *t*_*m*_, and those for *n* secondary cases are $\text{x}\prime =\left\{x_{1}^{\prime },x_{2}^{\prime },\ldots ,x_{n}^{\prime }\right\}$ and $\text{t}\prime =\left\{t_{1}^{\prime },t_{2}^{\prime },\ldots ,t_{n}^{\prime }\right\}$. Recall that *θ* = (*β*, *γ*, *λ*) is the vector of parameters that need to be estimated. Here *β* is the transmission rate of infection, and *γ*^−1^ is the mean infectious period, and *λ* is the mean degree. For the observed data, the likelihood function for *θ* can be constructed using (4) and (6):

$$L\left(\theta |\text{x},\text{t},\text{x}\prime ,\text{t}\prime \right)\propto \overset{m}{\underset{i=1}{\prod }}f_{\theta }\left(x_{i},t_{i}\right)\overset{n}{\underset{j=1}{\prod }}f_{\theta }\left(x_{j}^{\prime },t_{j}^{\prime }\right).$$

We could calculate the maximum likelihood estimates (MLEs) of *θ* by maximizing the expression above using numerical optimization. However, for better stability and reproducibility of results, we will use a Bayesian Markov Chain Monte Carlo (MCMC) procedure. We assign independent gamma prior distributions for the components of *θ*. Specifically, let

(8) $$\begin{array}{l} \pi (\beta )\sim \Gamma \left(a_{\beta },b_{\beta }\right)\\ \pi (\gamma )\sim \Gamma \left(a_{\gamma },b_{\gamma }\right)\\ \pi (\lambda )\sim \Gamma \left(a_{\lambda },b_{\lambda }\right). \end{array}$$

Given the data ***x***, ***t***, ***x*′**, and ***t*′**, the Bayesian estimate of *θ* is the mean of the joint posterior distribution

(9) $$L^{p}\left(\theta |x,t,x^{\prime },t^{\prime }\right)\propto L\left(\theta |x,t,x^{\prime },t^{\prime }\right)\pi (\beta )\pi (\gamma )\pi (\lambda ).$$

This mean may be approximated with an empirical average from the following converged MCMC sampler:

**Table:** 

Algorithm 1 MCMC posterior sampler for the branching process model
Initialize *θ* with *θ*_0_ = (*β*_0_, *γ*_0_, *λ*_0_). Sample *β* via a Metropolis-Hastings step [53] with truncated normal proposal for the target conditional distribution of *β* given ***x***, ***t***, ***x*′**, ***t*′**, *λ*. Sample *γ* directly from its conditional distribution $\gamma |\text{x},\text{t},\text{x}\prime ,\text{t}\prime \propto \gamma^{m+n+a_{\gamma }-1}e^{-\gamma \left(\sum^{\Sigma }t_{i}+\sum^{\Sigma }t_{j}^{\prime }+b_{\gamma }\right)}.$ Sample *λ* via another Metropolis-Hastings step (similar to Step 2) for the target conditional distribution of *λ* given ***x***, ***t***, ***x*′**, ***t*′**, *β*. Return to Step 2 and repeat until convergence.

### B. Survival model

Under the survival model, it is assumed that the population at risk (which is possibly much smaller than the set of all initially susceptible individuals) interacts homogeneously according to the Kermack-McKendrick model (see below). Hence it is problematic to apply this model in the case of an ETC where the homogenous mixing is likely not satisfied. Consequently, we apply this model only to the community outbreak data. In what follows it is convenient to write the usual Kermack-McKendrick model in the following integral form:

$$s_{t}=\text{exp}\left(-\beta \int_{0}^{t}\iota_{u}du\right)=\text{exp}\left(-R_{0}r_{t}\right)\;\iota_{t}=\rho e^{-\gamma t}-\int_{0}^{t}s_{u}e^{-\gamma (t-u)}du\;r_{t}=\gamma \int_{0}^{t}\iota_{u}du$$

where *ρ* = *l*_0_, and $R_{0}=\beta /\gamma$. By interpreting the strictly decreasing function *s*_*t*_ as an *improper survival function*, it follows [18] that the conditional density of infection time is given by

(10) $$f_{\tau }(t)=-{\dot{s}}_{t}/\tau$$

where *τ* = lim_*t*→∞_ *r*_*t*_ < 1 is the final size of the epidemic [18], which is the unique solution of

(11) $$1-\tau =e^{-R_{0}(\tau +\rho )}.$$

Thus, for a collection of *n* individuals initially at risk, out of which *k* are infected at respective times *t*_1_ <…< *t*_*k*_ < *T* (where *T* < ∞ is the maximum follow-up time), we have the following log-likelihood function for infection times

$$l_{I}\left(t_{1},\ldots t_{k}|\theta ,n\right)=(n-k)\text{log }s_{T}+\overset{k}{\underset{i=1}{\sum }}\text{log }f_{\tau }\left(t_{i}\right).$$

Note that this likelihood is conditional on the number of individuals at risk, which is often unknown. However, given the number of infections (*k*) by time *T* and under the assumption of independence of infection times (see [18] for discussion), we may consider *n* as the realization of the negative binomial distribution: *n* ~ NegBinom(*k*, *τ*). Denote by *w*_*i*_ the *i*th individual’s *removal time*, defined as minimum of the times of individual’s recovery, hospitalization, or death. Assuming *r* recoveries given *k* infections, the log-likelihood is

$$l_{R}\left(w_{1},\ldots ,w_{r}|\theta ,k\right)=(k-r)\text{log }H_{\gamma }(T)+\overset{r}{\underset{i=1}{\sum }}\text{log }h_{\gamma }\left(w_{i}\right),$$

where *H*_*γ*_(*·*) is the survival function and *h*_*γ*_(·) is the PDF of an exponential distribution with rate *γ*. The complete log-likelihood is then the sum of *l*_*I*_ and *l*_*R*_ [18]:

$$l_{0}\left(t_{1},\ldots ,t_{k},w_{1},\ldots ,w_{r}|\theta ,n\right)=l_{l}\left(t_{1},\ldots ,t_{k}|\theta ,n\right)+l_{R}\left(w_{1},\ldots ,w_{r}|\theta ,k\right).$$

Under this survival model, we may estimate the model parameters and the size of the population at risk *n* using another Bayesian MCMC. The model parameters are now *θ*′ = (*β*, *γ*, *ρ*), because *τ* is fully determined by *θ′* via (11). The prior distributions for *β* and *γ* are of the form (8) and the prior distribution for *ρ* is taken as Uniform(0, 1). All parameters are assumed independent a priori. The MCMC algorithm used to obtain the posterior sample of the parameters is similar to that in previous section.

**Table:** 

Algorithm 2 MCMC posterior sampler for the survival model
Initialize *θ*′ = (*β*, *γ*, *ρ*) from the prior distribution and set *n* = *k*. Perform a Metropolis-Hastings step (using the truncated normal proposal) for the target conditional distribution of *θ*′ given *n* using the complete log-likelihood *ℓ*_0_ = *ℓ*_*I*_ + *ℓ*_*R*_. Calculate *τ* based on the current value of *θ*′ as the solution to final size equation (11). Sample the conditional distribution of n given *θ*′ by drawing *n* ~ NegBinom(*k*,*τ*). Return to Step 2 and repeat until convergence.

## III. Data Analysis

The analysis was conducted separately for the two models (branching process and survival) based on the 48 available cases of EVD described in Section 1. Parameter estimates were obtained using the MCMC algorithms for each model described in Section 2. For the branching process model, we separately analyzed the community and ETC outbreaks. For the survival model, we only analyzed the community outbreak.

### A. Branching process model

To perform the separate analyses of the community and ETC outbreaks, the contact and infection data were partitioned into two subsets depending on the location of the infective contact (community or ETC). Although the same uninfected individuals were allowed to be in both outbreak networks, all the EVD cases were assigned either to the community or to the ETC. When reconstructing the transmission network, all ambiguous contact tracing was resolved uniformly at random as shown in Figure 1(b). For some individuals, the complete infection period was unknown and needed to be imputed. All such imputations were based on the density (2) and performed between steps 3 and 4 in MCMC algorithm.

For estimating *β* and *γ*, we assigned non-informative gamma prior distributions with location and rate parameters of 0.001. However, due to limited contact information, we assigned a relatively informative prior to λ with location parameter of 6 and a rate parameter of 1. This assignment was based on the empirical mean of the primary and secondary cases in the dataset. Finally, for the Metropolis-Hastings steps in the MCMC sampler algorithm we used the truncated normal distribution as proposal distribution and tuned its standard deviation to achieve an acceptance ratio of 44% as recommended in [54]. The final results of the MCMC were based on 55,000 iterations of the sampler with first 5,000 iterations removed as “burn-in”. The posterior samples were thinned by keeping only the results from every 10th iteration, resulting in a final set of 5,000 posterior samples that were used to estimate the parameters and calculate approximate posterior credible intervals. The convergence of the MCMC algorithm was diagnosed based on the *R* statistic and trace and autocorrelation (ACF) plots. To conserve space, these plots are not shown here.

Table I summarizes the results of branching process analyses of community and ETC epidemics. For each parameter, the posterior mean, standard deviation, and 95% credible interval (CI) are provided. We note that the comparisons between parameters in the two epidemics may be conducted informally by comparing their respective CIs bounds. If a particular parameter’s CI bounds for the community are contained within the respective CI bounds for the ETC, or vice-versa, one would consider the corresponding posterior distributions as statistically (i.e., for given data) equal. For *β*, which represents the transmission rate of Ebola virus, the posterior mean for household infection was approximately 0.0741—about twice as large as ETC infection rate—so the two posterior distributions may be considered statistically different. This is in contrast with the parameter *γ*, which represents the reciprocal of the mean infectious period, for which the estimated respective posterior means of 0.1936 and 0.2205 for the community and ETC were not found to be statistically different based on their respective 95% credible bounds. The parameter *λ* represents the average degree of the degree distribution and its posterior mean in the ETC is slightly (but not significantly) larger that its posterior mean in the community. This may reflect additional contacts of the individuals at the ETC with patients, visitors, and ETC staff. Finally, the posterior means of the basic reproduction numbers $R_{0}$ for the community and ETC outbreaks were found to be significantly different at 1.373 and 0.8592, respectively. As expected, the posterior mean of $R_{0}$ for the community outbreak is higher than one for the ETC. In both settings, $R_{0}$ was calculated according to equation (7). However, we also note that the 95% CI bounds for both posterior distributions are quite wide, indicating a lack of precision in the branching process model.

### B. Survival model

Under the survival model outlined in Section II-B, the analysis simplifies in that we are no longer concerned with estimating the network average degree *λ*. Hence our model parameters are *θ*′ = (*β*, *γ*, *ρ*) as well as the size of the population at risk (or effective population size) denoted by *n*. In this model, $R_{0}=\beta /\gamma$. The results of the analysis for the 37 community cases are presented in Table II. We note that the estimates of the parameters $\left(\beta ,\gamma ,R_{0}\right)$ under the survival model can be compared with the estimates of $\left(\beta \lambda ,\gamma ,R_{0}\right)$ under branching process model. Despite considerable differences in their respective posterior mean values, the posterior distributions of all three parameters are statistically equivalent based on the respective 95% CI bounds. This underscores the lack of precision of the estimates based on the branching process model.

### C. Model validation

**Fig. 2:**
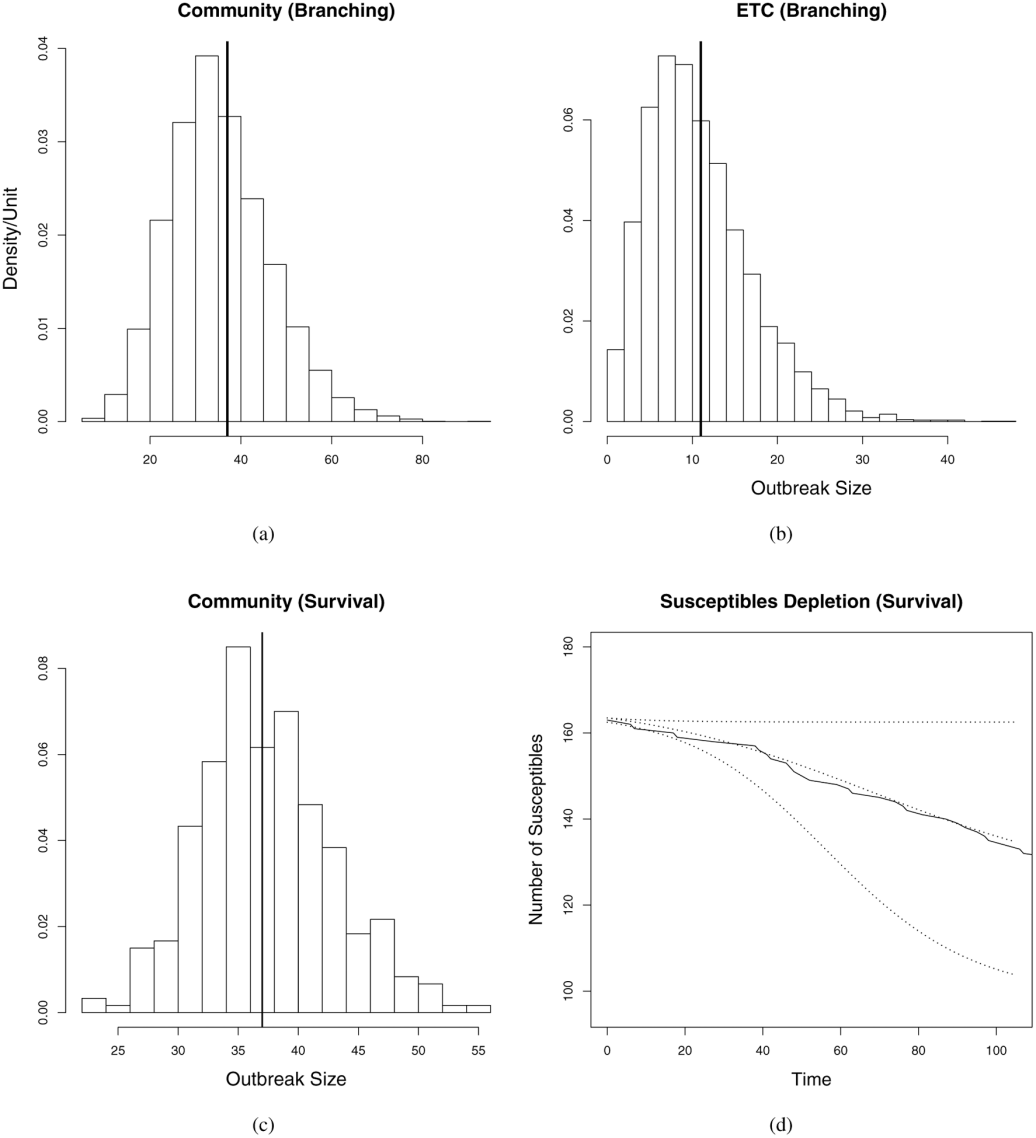
Model validation. Top panels: branching process model predicted final size distributions and observed values (marked with vertical lines) for the (a) community and (b) ETC outbreaks. Bottom panels: (c) survival model predicted final size distribution and the observed value and (d) survival model predicted depletion of the susceptible population and the observed depletion. The curves in (d) are drawn conditionally on the estimated mean initial population size *n* = 163 with the lower and upper dotted lines representing model’s 95% CI bounds.

To perform our model validation and fit assessment, we compared the distributions of 5,000 samples from the posterior distributions of the final outbreak size obtained from our branching process analysis to the values of 37 and 11 observed in the community and ETC outbreaks respectively. Figure 2 presents the histograms of the posterior size distributions for the respective final sizes. The vertical lines are plotted for reference at the observed outbreak values of 37 and 11. The histogram plots show that the observed values are close to the modes of the respective posterior distributions for both models, indicating adequate model fit [50]. The last panel of Figure 2 presents a comparison of observed depletion of susceptibles over the course of the community EVD outbreak with that predicted by the survival model. Both the observed and predicted curves are initiated on day one at the estimated mean of total population at risk. The two curves are very close to each other, indicating good fit of the survival model.

## IV. Summary and Conclusions

We presented two statistical models for analyzing patterns of EVD transmission in a small community. The branching process model was based on partial contact network tracing data, whereas survival model used an aggregate (network-free) approach. The two models allowed for a more detailed analysis of the 2012 Isiro EVD epidemic data that was summarized in [49].

The branching process model was based on a configuration model random graph with a Poisson degree distribution, and it explicitly described the direct and indirect contacts of the EVD cases in the community and at the ETC. In particular, the model made it possible to derive and directly compare the characteristics of the EVD Isiro outbreaks at these two different locations. Although the comparison provided some evidence of the usefulness of the ETC in controlling EVD outbreaks, the analysis also suggests that the type of basic contact tracing performed in Isiro may not be sufficient to provide precise estimates of the epidemic force via the basic reproduction number $\left(R_{0}\right)$.

The survival model was derived from the standard SIR equations. Since this model did not require contact tracing or estimation of mean degree, it did not suffer from the same problem of low precision as the branching process model. In fact, the estimate of $R_{0}$ provided by the survival model was more precise and likely also more accurate (based on a resampling analysis not shown here) than the values obtained from the branching process model. However, the drawback of the survival model was that it could not be used for comparison of transmission in the community and the ETC because the ETC was unlikely to satisfy the homogeneous mixing assumption.

It appears that an effective approach to modeling the type of outbreak dynamics described by the Isiro data might be to combine the two models presented here, so as to retain the precision of the survival one but also incorporate the transmission network information. This will be likely the focus of our future research.

## Figures and Tables

**TABLE I:** Results under the EVD branching process model for the community and ETC outbreaks

	Community infections			ETC infections		
	Mean	Std Dev	95% CI	Mean	Std Dev	95% CI
*β*	0.0741	0.0389	(0.0331, 0.1806)	0.0387	0.0182	(0.0152, 0.0851)
*γ*	0.1936	0.0397	(0.1254, 0.2811)	0.2205	0.0634	(0.1170, 0.3605)
*λ*	5.4460	1.4460	(2.9690, 8.6310)	5.9030	1.4200	(3.4200, 8.9820)
$R_{0}$	1.3730	0.2951	(0.8510, 2.0230)	0.8592	0.3200	(0.3700, 1.6040)

**TABLE II:** Results under the EVD survival model for the community outbreak only

	Mean	Std Dev	95% CI
*β*	0.1964	0.0324	(0.1403, 0.2626)
*γ*	0.1774	0.0296	(0.1258, 0.2381)
*ρ*	0.0039	0.0017	(0.0017, 0.0079)
*n*	163.20	35.35	(113.00, 252.00)
$R_{0}$	1.1080	0.0316	(1.0570,1.1650)

## Footnotes

1 We note here a slight discrepancy with [49] where only 34 cases were classified as community.
